# Coping with the Stress through Individual and Contextual Resilient Factors in Primary School Settings

**DOI:** 10.3390/bs13110880

**Published:** 2023-10-24

**Authors:** Raquel Flores-Buils, Clara Andrés-Roqueta

**Affiliations:** Department of Developmental, Educational Social and Methodological Psychology, Universitat Jaume I, 12071 Castellón de la Plana, Spain; candres@uji.es

**Keywords:** school stress, stressful situations, primary education, resilience, children, individual resilient factors, contextual resilient factors, school settings

## Abstract

Children face school stress as students through all educational stages. A negative association between resilience and stress has been demonstrated by many authors, but most of these studies have been carried out in higher educational stages. So, the aim of the present study is, on the one hand, to find out the level of stress of primary school children and also the types of stressful situations in school settings, and on the other hand, to analyze the effect of individual and contextually resilient factors on their level of school stress. The study involved 427 children between 6 and 12 years of age, who were administered the IECI school stress scale and the RES-PRIM Resilience questionnaire for children. Descriptive analyses, correlations, and regression analyses were performed on the data. Results showed an average level of school stress, with the most stressful situations being: participating in too many activities, concentration problems, and nervousness when being asked by the teacher in class. Predictive analysis showed that part of the school stress could be explained by both individual factors (self-esteem, introspection, future purpose, and social skills) and resilient contextual factors (teacher support, parental support, and peer support). It is concluded that it is necessary to pay more attention to the transitions between different educational stages with programs that reinforce academic information and encourage the development of individual resilient skills, stressing the importance of the role of teachers, peers, and parents as support groups.

## 1. Introduction

School stress can be defined as the relationship between the student and the demands of the academic environment, perceived by the student as threatening and endangering their well-being [1]. Across the different educational levels, students face various tasks and demands that require a large sum of both psychological and physiological adaptation skills, such as exams, competitiveness, delivery of deadlines, and work exhibitions, among others [2,3]. Therefore, school stress has been conceptualized as an adaptive and psychological process that involves the development of coping responses in students to face the conditions of academic life and that also promotes the appearance of symptoms of imbalance [4].

Specifically, academic stress appears when students feel they are not being able to cope with the demands of school activities or situations, and therefore they experience emotional tension [5]. In this sense, Gadzella and Masten [6] described the existence of both academic stressors (feelings of frustration, involvement in the conflict, pressure, change, and self-imposed) and reactions to academic stressors (physiological or physical, emotional, behavioral, and cognitive evaluations).

The presence of high or continuous stress can lead to poor academic performance [7], school dropout [8], poor sleep quality [9], depressive problems [10], tendencies to academic procrastination [11], social and contextual maladjustment, anticipatory anxiety about stressful events [12,13], and even suicidal ideation [14]. However, moderate or episodic school stress can improve academic performance [15], task performance [16], and productivity [17].

In this regard, several studies point out that students’ ability to cope with stress has a positive impact on their performance [18,19]. Thus, although chronically experienced stress can lead to physical, mental, and emotional problems, when the stress appears episodically, it can also negatively affect students’ cognitive performance because of their lack of coping [20]. Therefore, it is very important to detect stressful situations in school settings and to develop methods to improve students’ coping skills.

Mostly, there are two types of coping strategies: active and stressor-avoidance ones. Concretely, active strategies are those in which people make adjustments, changing their thinking or behavior to deal with stressors, and consequently enabling them to adapt themselves to stressful situations [21]. In this sense, the concept of resilience is important, as it is a quality that allows a person to prosper in the face of adversity. Thus, resilience is understood as the ability of people to adjust themselves, adapt, and even grow when it comes to face various difficult conditions (such as changes, demands, and feelings of disappointment or dissatisfaction that arise in life) [22]. In this way, resilient people are able to cope well with problems, control themselves, and manage stress by changing their way of thinking when facing problems [23].

It is important to highlight that resilience refers to an adaptive response that is activated when a person faces difficult conditions [24]. So, having resilient skills does not imply not experiencing these situations as difficult, but knowing how to manage, confront, and overcome them [25]. In this regard, resilience can be modulated by risk and protective factors. On the one hand, risk factors represent individual and environmental variables that increase the probability of negative responses in adverse situations. On the other hand, protective factors are a set of subject and context variables that enhance the capacity to resist conflict and manage stress. The real effect of these factors is manifested when the risk is present, and they act as compensators (modulation, decrease, or elimination) [26,27].

During childhood, Werner [27] considers that resilience in children has three main components: individual and contextual variables (mainly, family structure and extrafamilial context). Among the child’s individual variables, the author highlights self-esteem, introspection, empathy, and problem-solving capacity. Among the contextual variables, the author points out the supportive relationships with peers and the security and protection systems of adults within the family, school, and social environment. In this respect, the family and school exert a clear protective role when they exhibit affection, cohesion, openness, commitment, support, and positive role models, and they ensure the absence of risk factors [28]. According to resilience theory, an individual’s resilience is determined by the balance between these individual and contextual factors in the face of adversity [29]. Therefore, it can be said that individuals who have high resilience have a greater ability to better adapt themselves to their environment [5].

### Theoretical Background

School stress is a phenomenon that is present today and affects the educational community in general [30]. Thus, a study conducted by the Organization for Economic Co-operation and Development (OECD) surveyed more than 500,000 students aged 15–16 years in 72 countries. The results indicate that more than 60% of the respondents felt stressed and anxious for academic reasons such as low marks or evaluation situations [8]. These data agree with other studies conducted with middle school students [31,32]. Regarding elementary education, a study found that 54% of children also felt stressed in school situations [33].

Regarding variables such as gender and age of students, many studies have found that girls perceive greater school stress than boys [34,35,36]. Regarding their age, studies suggest that the older the student is, the greater the use of coping strategies is, which can be translated into better management of school stress [37]. Moreover, several authors point out that the most stressful situation is the change of educational stage, the uncertainty and adaptation to it, since for many children it means a change of center, with new classmates and teachers, and new methodologies [38,39,40,41].

Associations between school stress and resilience have also been explored. Several studies have shown that resilience is negatively associated with school stress [42], because students with high resilience experienced less stress and fewer psychosomatic symptoms in the face of exams and situations of academic load or pressure. Moreover, highly resilient adolescents have been seen to attend school and participate in school activities more actively than their less resilient peers [43].

Despite the prevalence and relevance of school stress in primary school settings, studies on school anxiety have focused mainly on adolescents [44,45] and the university environment [46], and there are few studies that pay attention to school stress and the association with resilience during primary education. If these stressful situations already occur in primary education, and taking into account that resilience can be promoted, it is important to deal with stressful situations from this stage and develop methodologies to promote resilient skills before they move on to high school/university and encounter transitions and situations that may be much more stressful and do not have these strategies available. Therefore, the aim of the present study is to analyze the degree of school stress and types of stressful situations in primary school children, as well as the effect of individual and contextual resilience factors on the level of school stress in these students.

Based on the existing literature, we propose the following hypotheses:

**Hypothesis** **1.** *Younger children attending lower courses through primary school, above all girls, will show higher school stress levels;*

**Hypothesis** **2.** *The types of situations perceived as more stressful will vary according to the course within primary school;*

**Hypothesis** **3.** *Resilience, as well as all individual and contextual factors, will be negatively associated with school stress;*

**Hypothesis** **4.** *Both resilient individual and contextual factors will explain school stress.*

## 2. Materials and Methods

### 2.1. Participants

A total of 427 primary school students aged between 6 and 12 years (M = 8.89; SD = 1.788) who attended different public schools in Valencian County (Spain) participated in the present study. 51.75% were boys and 48.24% were girls. Table 1 shows the number and percentages of participants broken down by grade and sex.

### 2.2. Instruments

School Stress Scale of the Everyday Childhood Stress Inventory (IECI) [47]. This scale reports on children’s perceived stressors related to school situations. Children are asked if they find schoolwork difficult, if they usually get bad grades, if teachers are very demanding of them, if they participate in too many extracurricular activities, if they find it hard to concentrate on homework, if they get nervous when asked by the teacher, or if their classmates pick on them at school. It consists of 7 dichotomous response items, and it is intended to be administered to children between 6 and 12 years old. The score ranges from 0 to 7. The scale obtained good psychometric properties in the original study (α = 0.81) [47] and also in our study (α = 0.81). IECI represents a brief self-report instrument, easy to administer and answer, that has shown adequate psychometric properties and has been used in multiple studies analyzing children’s stress [48,49,50].

Resilience Scale for Primary School Students (RES-PRIM) [51]. This scale informs children’s resilient skills. It consists of 38 items, of which 24 measure individual resilient factors (empathy/prosociality; social skills; self-esteem; self-awareness and emotional regulation; problem solving and future/life purpose); and 14 measure protective contextual factors (teacher support; parent support; peer support). Each item presents a Likert-type response scale (0 = never, 1 = many times, 2 = few times, 3 = always), where each participant has to indicate to what extent each of the statements has been true for him/her during the last month. Total scores range from 0 to 114 for the total scale (0–72 for the individual resilient factors subscale and 0–42 for the contextual resilient factors subscale), where a higher score expresses greater resilience. The scale, in the final phase of validation, is designed for children aged between 6 and 12 years old, and it has good psychometric properties (α = 0.86). In our study, this scale has also obtained good reliability (α = 0.82).

### 2.3. Procedure

First, the corresponding permissions were requested from the autonomous government of Valencian County. In addition, this study was approved by the Ethics Committee of the university to which the authors belonged. Then, the researchers explained the aims of the study to the selected primary schools. After their approval, individual informed consent was requested from the students’ parents.

Finally, the research group went to each of the different schools to administer the instruments to the students. The questionnaires were administered in the second semester of the course, and on the same day, both questionnaires were passed. The centers provided the research group with a classroom that was available and equipped for the administration of instruments. In the first and second grades, the instruments were administered individually by a member of the research group to each child due to the level of reading comprehension of the students in these courses. From the third to the sixth grade, the administration of both instruments was carried out collectively in each class with the supervision of two members of the research group. The two questionnaires were the same for all courses. For the collective pass, the two paper questionnaires were distributed to each child. One of the researchers presented the questionnaires to the children, and she read the items aloud and slowly so that the children could answer them. Additionally, there were two more members of the research team in the classroom for support in case any child had any questions.

### 2.4. Data Analysis

Statistical analysis was performed with the SPSS 29.0 program. First, the Kolmogorov-Smirnov test was performed. The data were found to have a normal distribution (Z = 0.161; *p* = 0.12).

Then, descriptive analyses of school stress (mean, standard deviation, and percentages) were carried out, taking into account gender and course of the children. Moreover, Student’s *t*-test for independent samples was also performed to analyze whether there is a difference in school stress according to the sex of the participants, and an analysis of variance (ANOVA) was performed to analyze whether there are differences in school stress according to grade. The corresponding effect sizes (Cohen’s *d* and Eta squared) were calculated. Then, Pearson correlations were performed to find out the association between school stress and resilience (total, individual resilient factors, and contextual resilient factors).

Finally, a multiple linear regression was performed to find out which individual and contextually resilient factors predict a greater development of stress in primary school children.

## 3. Results

Table 2 presents the mean and standard deviation of the level of school stress, as well as the percentage of students who perceive the different situations measured as stressful, taking into account the sex and age of participants. All these data are shown both for the total sample and for the six grades of primary education.

The levels of school stress shown in Table 2 show how in the first two grades the level of school stress is higher, and from the third grade onwards the values decrease. These values, both overall and by sex, correspond to average values. If we compare the values found with the scale scales calculated by age and sex, it is observed that the stress level of the children in this study corresponds to standard score (T) values of between 45 and 50 at all ages with respect to the data from the instrument scales [47]. Taking into account the scales, 45.4% of the children in our study would show no stress problems, 41.5% would show mild stress, and 13.1% of the students would show high stress.

In order to determine whether there are significant differences in the scores obtained in school stress according to the participants’ grade and sex, an ANOVA was performed. According to age, data indicate that there are significant differences (F = 5.705, *p* < 0.001, η^2^ = 0.59) in stress according to school year. Likewise, to analyze possible differences in children’s stress according to the sex variable, a comparison of means for independent samples was carried out. The data indicate that there are no significant differences (*p* = 1.99; d = 0.28), although it is found that the mean stress of girls (M = 2.81; SD = 1.486) is higher than that of boys (M = 2.70; SD = 1.517).

Regarding the type of stressful situations, as also shown in Table 2, the situations with the highest percentage in all grades are related to teachers’ demands; participating in too many extracurricular activities; having problems concentrating on a task; and getting very nervous when the teacher asks them questions in class.

Subsequently, in order to know the level of resilience of the children and its relationship with the level of school stress, the mean score obtained in resilience (total and by individual and contextual factors) and the correlations with the school stress variable are presented (Table 3).

Regarding resilience, the scores obtained show medium-high levels of resilience both on the total scale and in each of the resilience factors. Moreover, it is observed that there is a significant and negative correlation between total resilience and internal and contextual factors and school stress.

In a more detailed way, Table 4 shows the relationship between each of the individual resilient variables with school stress and with each of the stressful school situations.

As shown in Table 4, total school stress is negatively correlated with all individual resilient variables, i.e., empathy/prosociality, social skills, self-esteem, introspection, problem solving, and future purpose.

Regarding the relationship of each individual resilient factor with each of the stressful situations, the following negative correlations are observed: empathy/prosociality with homework difficulty, concentration problems, getting nervous when the teacher asks them; the social skills variable with concentration problems, getting nervous when the teacher asks them and when classmates pick on them; the self-esteem variable with all situations except teacher demand; the introspection variable with all situations except with getting bad marks and having concentration problems; the problem-solving variable with getting bad marks, having concentration problems and getting nervous when the teacher asks them; finally, the future purpose variable with concentration problems, getting nervous when the teacher asks them and when their classmates pick on them.

On the other hand, Table 5 shows the relationship between the different types of support that make up the resilient contextual factor, school stress, and each of the stressful school situations.

Regarding the relationship of each contextual variable with each of the stressful situations, the following negative correlations are observed: Teacher support with all stressful situations except when classmates pick on them; Parent support with participation in too many extracurricular activities and concentration problems in class; and peer support with the teacher’s demands, concentration problems, and getting very nervous when the teacher asks them questions in class and when classmates pick on them.

Finally, two multiple regressions were carried out in order to determine which aspects explain school stress in primary school children.

First, a multiple linear regression analysis was performed for school stress, introducing the individual resilient factor and the contextual resilient factor as independent variables. This model explained 21.3% of the variance: F = 34.8, R2 = 0.213, *p* < 0.001; and as shown in Table 6, the variable that most explained school stress was the individual resilient factors (*β* = −0.201), followed by the contextual resilient factors (*β* = −0.199), although the values were very similar. The variance inflation factor (IVF) values are less than 10 in all cases, and the tolerance (T) values are greater than 0.1, which indicates the non-existence of linear relationships between the independent variables.

Secondly, a multiple linear regression analysis was performed for school stress, introducing the different individual and contextual variables. This model explains 29.7% of the variance (see coefficients in Table 7): *F* = 16.051, *R*^2^ = 0.297, *p* < 0.001. In this case, the variable that most explained school stress was having peer support (*β* = −0.245), followed by the variables self-esteem (*β* = −0.198), introspection (*β* = −0.187), future purpose (*β* = −0.122), parental support (*β* = −0.116), teacher support (*β* = −0.107), and social skills (*β* = −0.103). Nevertheless, empathy/prosociality and problem-solving variables did not predict school stress. The variance inflation factor (IVF) values are less than 10 in all cases, and the tolerance (T) values are greater than 0.1, which indicates the non-existence of linear relationships between the independent variables.

## 4. Discussion

The aim of the present study was to analyze the degree of school stress and types of stressful situations in primary school children (taking sex and age into account) and also to analyze the effect of individual and contextual resilience factors on the level of school stress in these students.

First, hypothesis 1 stated that younger children attending lower courses through primary school, above all girls, would show higher school stress levels. Descriptive data confirmed an average level of school stress within the sample (6–12 years old). Regarding the age of the children, between-group comparisons taking into account the academic course/grade showed that younger students in the first two years have a higher mean of stress than those on later courses. This may be due to the fact that at the beginning of a stage, new situations often appear that can be stressful during the adaptation period [40]. In addition, the transition from preschool education to primary education involves the incorporation of situations that generally generate stress for all students, such as the incorporation of exams, changes in teachers and methodologies, a greater number of teachers, or homework to do at home. These results agree with previous studies [38,39,41], which lead us to support the idea that transitions between educational stages and adaptation to them are moments that generate greater stress for students. On the other hand, with respect to the sex of the children, no differences were found in the level of stress perceived by boys and girls, although the mean for girls was higher. Although the difference is not significant, this is in line with many other studies that have found significant differences in the level of school stress according to sex, and in all of them, girls have the highest level [41,52,53]. In this regard, there are studies that point out that this difference could be due to cultural patterns promoted in the processes of socialization in childhood and adolescence, since it is still possible to observe how girls express their feelings and emotional reactions more freely, unlike boys, who tend to show bravery and competitiveness [54].

Hypothesis 2 stated that the types of situations perceived as more stressful would vary according to the course within primary school grade. In this sense, the data from the present study does not confirm the prediction because the types of situations perceived as most stressful (teachers’ demands, participating in too many extracurricular activities, having problems concentrating on a task, and becoming very nervous when the teacher asks them questions in class) did not vary according to the school year of the children. Several studies have pointed out the existence of different stressful situations in the school context, which coincide to a greater or lesser extent with those analyzed in the present study: high workload or tasks inside and outside the educational establishment, teacher evaluations, competitiveness, fear of failure or not achieving self-imposed or externally stipulated goals, parental pressure, peer group acceptance, peer rivalry, among others [20,55,56]. Nevertheless, from the data obtained in the present study, it is important to highlight which concrete situations (among commonly stressful situations) are perceived as the most stressful among elementary school students, because it will help us to understand the students’ real experiences and will allow us to design more precise and effective prevention strategies.

Hypothesis 3 stated that resilience, as well as all individual and contextual factors, would be negatively associated with school stress. The correlational analysis raised in this study confirmed this negative association. Furthermore, significant negative correlations were obtained with both general and individual and contextual resilient factors, coinciding with previous studies [5,38,57,58,59].

Therefore, the role of resilience in coping with school stress is also confirmed for primary school students. In this sense, Table 4 and Table 5 present a more specific analysis of those individual and contextual variables that correlate with children’s school stress. Thus, with respect to the individual variables, it is observed that all of them are significantly related to school stress. Other similar studies have also found a relationship between school stress and individual variables such as self-esteem, self-concept, social skills, problem solving, and emotional development [37,52]. However, although the variable *introspection* has not been found as such in any study, it is closely related to self-awareness and emotional self-regulation, variables that have been analyzed in other studies in which a negative relationship between these variables and stress has been found. Thus, we would like to highlight the positive correlation observed between *empathy/prosociality* and *future purpose* with school stress, since these variables have been little studied in school settings. Specifically, existing studies focused on the relationship between empathy/prosociality and stress present mixed results: on the one hand, some studies demonstrated a negative relationship between both, because empathic people present greater mental flexibility, emotional self-regulation, communication, and self-awareness that leads to less stress [37]; but on the other hand, there are studies that point to the fact that a high degree of empathy can generate emotional exhaustion that would lead to greater stress [59,60], maybe because primary school children do not want to disappoint their parents and teachers so as not to make them feel bad. Therefore, it is important to continue analyzing this variable. Regarding the *future purpose* variable, different studies conducted in higher educational stages indicate that it represents a marked experiential intentionality, a clear idea of the meaning of life with an optimistic approach, a sense of commitment to problem solving and flexibility, and adaptation to situations, which in sum moderates academic stress [35,61]. Therefore, it is also important to continue analyzing this variable in the primary education stage.

Regarding resilient contextual factors, it is observed that the three types of perceived support (teachers, parents, and peers) correlate significantly and negatively with school stress. These data are in line with the findings of other studies that point to the importance of these social supports (family, school, and peers) as fundamental aspects of both school stress [28,30,33], and the development of children’s resilience [38,62]. In the present study, a detailed analysis has been conducted in order to find out in which situations each type of support is more significant for children (taking into account the types of stressful situations). In fact, it is curious to observe how parental support is only significantly related to extracurricular activities (possibly because parents are those who accompany them and even encourage them to go) and to the ability to concentrate (possibly because parents show them trust and understanding). In contrast, peer support is related to situations that occur within the classroom related to interaction with the teacher and with other classmates. It should be noted that teacher support is related to all situations, except for situations in which one classmate picks on another. So, these results support and emphasize that teacher support plays a key role in the wellbeing of students at this stage in the educational context.

Finally, with respect to hypothesis 4 of our study, which refers to the fact that individual and contextual resilience factors would explain school stress similarly, the regression analyses carried out showed that, with very similar weights, individual resilience factors explained school stress to a greater extent than contextual factors. This result would be supported by the concept of resilience itself, since the construction of resilience depends on the functioning of individual and contextual factors and their continuous interdependencies [63]. In this sense, the degree of resilience is determined by the balance between individual and contextual resilient factors in the face of an adverse or difficult situation [29].

Likewise, the predictive analysis carried out on each of the individual and contextually resilient aspects indicates that scholar stress would be explained by peer support, self-esteem, introspection, future purpose, parental support, teacher support, and social skills. So, this is an important contribution to existing literature, as it allows us to better understand school stress in primary school children and also to establish strategies for prevention and/or intervention.

## 5. Conclusions

The level of school stress is higher in children in the first years of primary school. Thus, although the types of school situations that most generate stress do not vary according to the grade, the level of perceived stress does.

In this regard, school stress is found to be negatively related to both individual and contextual resilient variables. Although there is a relationship with all of them, among the individual resilient factors, we would like to highlight the relationship with empathy/prosociality and future purpose, since these variables have been little studied in this educational stage. Among the contextual factors, we would like to highlight the importance of each support group depending on the type of stressful situation, with the teacher being the one who turns out to be a support in almost all school situations. Finally, it should be noted that individual resilient factors have a similar weight as contextual resilient factors in coping with stress.

Taking these contributions into account, we would like to emphasize the importance of reinforcing the actions that schools usually carry out in the first year to welcome new students at this educational stage. It would be necessary to work on the transition between stages from two perspectives. On the one hand, the academic aspect involves informing children during the last year of early childhood education and during the first weeks of primary education about what they will find in primary education (new methodologies, subjects, types of evaluation, number of teachers they will have, what happens if an exam goes wrong, what happens if they ask me something in class that I do not know, if they will know all their classmates or if they will be new, who they can talk to if they have a problem, among other aspects). This information would help them become familiar with the changes of the new stage and reduce the anxiety caused by uncertainty. On the other hand, it would be important to reinforce the integral development of students with intervention programs for the development of individual skills. These programs must include enhancement of empathy/prosociality, self-esteem, social skills, introspection, problem solving, and future purpose, together with stress management strategies and coping skills. To achieve this goal, the role of educational psychologists in primary schools is fundamental, both in the development of these aspects and in the detection of specific situations of high stress in the student body. In addition, these programs would be carried out in the class group and with active methodologies that facilitate students getting to know each other better, promote group cohesion, and create a better classroom climate.

These programs should be accompanied by manuals, guides, or training sessions for the parents of these students and the teachers who teach in these first years of primary education to be aware that for these children, they are their reference person in difficult or complex situations, a person who understands them, accepts them as they are, and encourages them to find themselves and to make sense of what they are experiencing in certain situations. This is especially important for those who suffer emotionally from stress. Adults close to these children can be someone who can offer them strategies that strengthen them and allow them to function in their environment, being fundamental for the creation of an adequate bond between these adults and children [64]. To this end, it is important to point out the role of teachers in favoring the development of resilience in students and generating new possibilities to solve the multiple and complex situations presented by the school context through daily teaching.

Finally, we would like to point out a series of limitations that should be considered in other studies. On the one hand, it would be interesting to extend the sample of children with students of the same stage but from other countries, as this would help to generalize the results. In addition, school stress could be analyzed in children with atypical development, such as children with neurodevelopmental disorders or other problems. Likewise, other types of tests to measure stress could be included, both quantitative and qualitative, which would help to better understand how children experience the situations they perceive as stressful. Thus, stress collected with self-report instruments gives us information on perceived stress, which could be complemented with psychobiological tests, such as the cortisol/DHEA ratio, by taking saliva samples from the students.

## Figures and Tables

**Table 1:** Description of the age and gender of participants.

Course	n	Aged	n Sex (%)	
		M (DE)	Boys	Girls
1st (6–7 years)	65	6.17 (0.382)	33 (50.7%)	32 (49.3%)
2nd (7–8 years)	72	7.27 (0.446)	39 (54.1%)	33 (45.8%)
3rd (8–9 years)	62	8.38 (0.488)	35 (56.4%)	27 (43.5%)
4th (9–10 years)	75	9.15 (0.357)	33 (44%)	42 (56%)
5th (10–11 years)	74	10.23 (0.420)	40 (54.05%)	34 (45.9%)
6th (11–12 years)	79	11.26 (0.440)	41 (51.8%)	38 (48.1%)
Total	427	8.89 (1.788)	221 (51.75%)	206 (48.24%)

**Table 2:** Mean and standard deviation of school stress, together with the percentage of stressful situations.

				*Stressful School Situations*						
Course	School Stress M (DS)			1	2	3	4	5	6	7
	Total (0–7)	Boys (0–7)	Girls (0–7)	%	%	%	%	%	%	%
1st	3.08 (1.51)	2.85 (1.61)	3.32 (1.38)	8.7	4.3	26.1	44.8	56.5	62.2	26.1
2nd	3.30 (1.38)	3.28 (1.32)	3.42 (1.48)	34.6	3.8	30.8	50	60	63.8	14.1
3rd	2.83 (1.36)	2.46 (1.25)	2.92 (1.41)	21	7.4	24.7	39.6	55.7	64.2	11.1
4th	2.5 (1.66)	2.37 (1.17)	2.65 (1.92)	17.2	9.4	19.6	44.4	47.5	65.3	10.9
5th	2.43 (1.62)	2.30 (1.28)	2.59 (1.59)	17.5	11.3	12.5	32.5	52.5	66.3	11.3
6th	2.35 (1.21)	2.16 (1.03)	2.56 (1.31)	3.2	6.5	16.1	48.7	45.5	61.9	12.9
Total	2.75 (1.5)	2.70 (1.51)	2.81 (1.48)	17	7.1	20.2	45.1	54.3	60.5	14.2

Note: 1. Difficulty with schoolwork; 2. Poor marks usually; 3. Teachers are very demanding; 4. Participating in too many extracurricular activities; 5. Problems concentrating on a task; 6. Get very nervous when the teacher asks me; 7. Classmates pick on me a lot.

**Table 3:** Mean, standard deviation, and correlations between school stress and resilience (total, individual, and contextual factors).

		*School Stress*	
	Mean (SD)	*r*	*p*
Resilience (0–114)	91.76 (11.46)	−0.361 **	<0.001
Individual resilience factors (0–72)	56.02 (7.31)	−0.933 **	<0.001
Contextual resilience factors (0–42)	35.74 (5.33)	−0.870 **	<0.001

Note: ** *p* < 0.01.

**Table 4:** Correlations between individual resilient factors and school stress (total and stressful situations).

	Empathy/ Prosociality		Social Skills		Self-Esteem		Introspection		Problem Solving		Future Purpose	
	*r*	*p*	*r*	*p*	*r*	*p*	*r*	*p*	*r*	*p*	*r*	*p*
SS	−0.200 **	<0.001	−0.092 *	0.047	−0.292 **	<0.001	−0.304 **	<0.001	−0.206 **	<0.001	−0.100 *	0.031
1	−0.251 **	<0.001	0.026	0.579	−0.144 **	0.002	−0.121 **	0.009	−0.084	0.072	−0.004	0.936
2	0.001	0.978	0.049	0.291	−0.122 **	0.008	−0.029	0.532	−0.097 *	0.036	0.015	0.742
3	0.012	0.795	−0.017	0.712	−0.016	0.729	−0.136 **	0.003	−0.069	0.139	−0.038	0.419
4	0.036	441	0.029	0.533	−0.115 *	0.013	−0.109 *	0.018	0.083	0.074	0.026	0.571
5	−0.199 **	<0.001	−0.132 **	0.004	−0.275 **	<0.001	−0.303	<0.001	−0.203 **	<0.001	−0.111 *	0.017
6	−0.215 **	<0.001	−0.141 **	0.002	−0.288 **	<0.001	−0.258 **	<0.001	−0.285 **	<0.001	−0.133 **	0.004
7	−0.079	0.090	−0.116 *	0.012	−0.171 **	<0.001	−0.128 **	0.006	−0.091	0.050	−0.123 **	0.008

Note: ** *p* < 0.01; * *p* < 0.05; SS—school stress; 1. Difficulty with school work; 2. Poor marks usually; 3. Teachers are very demanding; 4. Participating in too many extracurricular activities; 5. Problems concentrating on a task; 6. Get very nervous when the teacher asks me; 7. Classmates pick on me a lot.

**Table 5:** Correlations between the resilient contextual factors and school stress (total and stressful situations).

	Teacher Support		Parent Support		Peer Support	
	*r*	*p*	*r*	*p*	*r*	*p*
SS	−0.225 **	<0.001	−0.100 *	0.032	−0.326 **	<0.001
1	−0.121 **	0.009	0.056	0.224	−0.077	0.097
2	−0.137 **	0.003	0.024	0.611	−0.128	0.006
3	−0.296 **	<0.001	0.018	0.703	−0.235 **	<0.001
4	−0.125 **	0.007	−0.192 **	<0.001	−0.079	0.090
5	−0.099 *	0.032	−0.136 **	0.003	−0.205 **	<0.001
6	−0.139 **	0.003	−0.062	0.181	−0.231 **	<0.001
7	−0.013	0.779	−0.105	0.023	−0.322 **	<0.001

Note: ** *p* < 0.01; * *p* < 0.05; SS—school stress; 1. Difficulty with school work; 2. Poor marks usually; 3. Teachers are very demanding; 4. Participating in too many extracurricular activities; 5. Problems concentrating on a task; 6. Get very nervous when the teacher asks me; 7. Classmates pick on me a lot.

**Table 6:** Regression coefficients of individual and contextual resilience factors for school stress.

Predictor	B	SE B	*β*	t	*p*	T	VIF
(Constant)	6.079	0.527		11.540	<0.001		
Individual resilience factors	−0.041	0.012	−0.201	−3.585	<0.001	0.598	1.673
Contextual resilience factors	−0.056	0.016	−0.199	−3.553	<0.001	0.615	1.865

**Table 7:** Regression coefficients of the individual and contextually resilient variables significantly correlated with school stress.

Predictor	B	SE B	*β*	t	*p*	T	VIF
(Constant)	6.550	0.576		11.340	<0.001		
Empathy/Prosociality	−0.065	0.035	−0.086	−1.855	0.064	0.808	1.237
Social skills	0.138	0.063	−0.103	−2.180	0.030	0.787	1.270
Self-esteem	−0.220	0.054	−0.198	−4.091	<0.001	0.742	1.347
Introspection	−0.086	0.024	−0.187	−3.593	<0.001	0.647	1.546
Problem solving	−0.015	0.048	−0.015	−0.303	0.762	0.717	1.395
Future purpose	0.098	0.038	−0.122	−2.552	0.011	0.767	1.304
Teacher support	−0.082	0.036	−0.107	−2.269	0.024	0.787	1.271
Peer support	−0.104	0.022	−0.245	−4.463	<0.001	0.632	1.581
Parent support	0.116	0.049	−0.116	−2.389	0.017	0.741	1.349

## Data Availability

The data presented in this study are available upon request from the corresponding authors.
